# Relevance of Copy Number Variation at Chromosome X in Male Fetuses Inherited from the Mother May Be Ascertained by Including Male Relatives from the Maternal Lineage in Addition to Trio Analyses

**DOI:** 10.3390/genes11090979

**Published:** 2020-08-22

**Authors:** Ming Chen, Wan-Ju Wu, Mei-Hui Lee, Tien-Hsiung Ku, Gwo-Chin Ma

**Affiliations:** 1 Department of Genomic Medicine and Center for Medical Genetics, Changhua Christian Hospital, Changhua 50046, Taiwan; 104060@cch.org.tw (M.C.); crystalwu835@gmail.com (W.-J.W.); 29561@cch.org.tw (M.-H.L.); 2 Department of Obstetrics and Gynecology, Changhua Christian Hospital, Changhua 50006, Taiwan; 3 Research Department, Changhua Christian Hospital, Changhua 50006, Taiwan; 4 Department of Genomic Science and Technology, Changhua Christian Hospital Healthcare System, Changhua 50046, Taiwan; 5 Department of Medical Genetics, National Taiwan University Hospital, Taipei 10041, Taiwan; 6 Department of Obstetrics and Gynecology, College of Medicine, National Taiwan University, Taipei 10041, Taiwan; 7 Department of Biomedical Science, Dayeh University, Changhua 51591, Taiwan; 8 Department of Medical Science, National Tsing Hua University, Hsinchu 30013, Taiwan; 9 Ph.D. Programs in Translational Medicine, National Chung Hsing University, Taichung 40227, Taiwan; 10 Department of Anesthesiology, Changhua Christian Hospital, Changhua 50006, Taiwan; 11 Department of Biomedical Engineering, Chung Yuan Christian University, Taoyuan 32023, Taiwan; 12 Department of Medical Laboratory Science and Biotechnology, Central Taiwan University of Science and Technology, Taichung 40601, Taiwan

**Keywords:** microarray, chromosome X, CNV, prenatal diagnosis, genetic counseling

## Abstract

Chromosome microarray analysis has been used for prenatal detection of copy number variations (CNVs) and genetic counseling of CNVs has been greatly improved after the accumulation of knowledge from postnatal outcomes in terms of the genotype-phenotype correlation. However, a significant number of CNVs are still regarded as variants of unknown significance (VUS). CNVs at the chromosome X (X-CNVs) represent a unique group of genetic changes in genetic counseling; X-CNVs are similar to X-linked recessive monogenic disorders in that the prognosis in males is expected to be poor. Trio analysis is typically advised to patients with X-CNVs but such an approach may be inadequate in prenatal settings since the clinical relevance is sometimes uninformative, particularly for the maternally inherited X-CNVs in male fetuses. Here, we reported four healthy women whose male fetuses were found to have X-CNVs inherited from the mothers. The X-CNVs were initially recognized as VUS or likely pathogenic in males according to the publicly available information. After extending genetic analyses to male relatives of the maternal lineages, however, the relevance of the X-CNVs was reconsidered to be likely benign. The results highlight that an extended analysis to include more relatives, in addition to the parents, provides further information for genetic counseling when X-CNVs are encountered in prenatal settings.

## 1. Introduction

Over the past decade, chromosome microarray analysis (CMA) has been increasingly used in routine prenatal examinations because it enables a genome-wide characterization of submicroscopic deletions and duplications, known as copy number variants (CNVs), and improves the diagnostic yields of rare diseases [1]. However, CNVs do not always reflect adverse phenotypes. It is known that approximately 9.5% of the healthy human genome contributed to CNVs, with a mean size of 341 kilobase pairs (kb) [2,3]. Interpretation of CNVs in fetuses is thus challenging because the genotype-phenotype relationship remains inconclusive in certain conditions and in such cases the variant is commonly referred to as variant of unknown significance (VUS). In prenatal settings, multiple lines of information (e.g., findings of fetal anatomy by image examinations, features of previously reported cases with similar CNVs, results of parental follow-up analysis, as well as the sizes and genes included of CNVs) must be taken into consideration while prospecting the clinical consequences of CNVs [4]. Moreover, when dealing with CNVs on chromosome X (X-CNVs), the gender information must be also considered. A higher risk is expected in males than females since males only have one chromosome X and genomic imbalances of the chromosome X can cause abnormal consequences. On the contrary, female carry one copy of X-CNV are seldom affected except under a few conditions, such as skewed X chromosome inactivation (XCI) [5]. The differences between male and females in risk and outcome, making X-CNVs distinct from the CNVs on autosomes [6,7]. Trio fetus-mother-father analysis is typically advised to patients with X-CNVs but such approach may be inadequate in prenatal settings since the clinical relevance is sometimes uninformative, particularly for the maternally inherited X-CNVs in male fetuses.

In the present study, we reported four healthy women (from four families) who were informed about having transmitted the maternal X-CNVs to the male fetuses when they underwent prenatal CMA. The X-CNVs were initially considered to be VUS or likely pathogenic according to the public databases and published reports. However, pedigree analysis demonstrated that all the X-CNVs can be found in healthy male relatives of the maternal lineages (e.g., grandfather or uncle) in these families, rendering the X-CNVs as likely benign. We highlight that an extended study, in addition to the trio analysis, including male relatives of the maternal lineage provides additional information for prenatal counseling when male fetuses carried X-CNVs from their heathy and asymptomatic mothers.

## 2. Materials and Methods

### 2.1. Patients

This work is a retrospective medical record review study which is proved by Institutional Review Board of Changhua Christian Hospital, Taiwan (Project No.: 200408; approval date: 7 May 2020).

**Figure 1:**
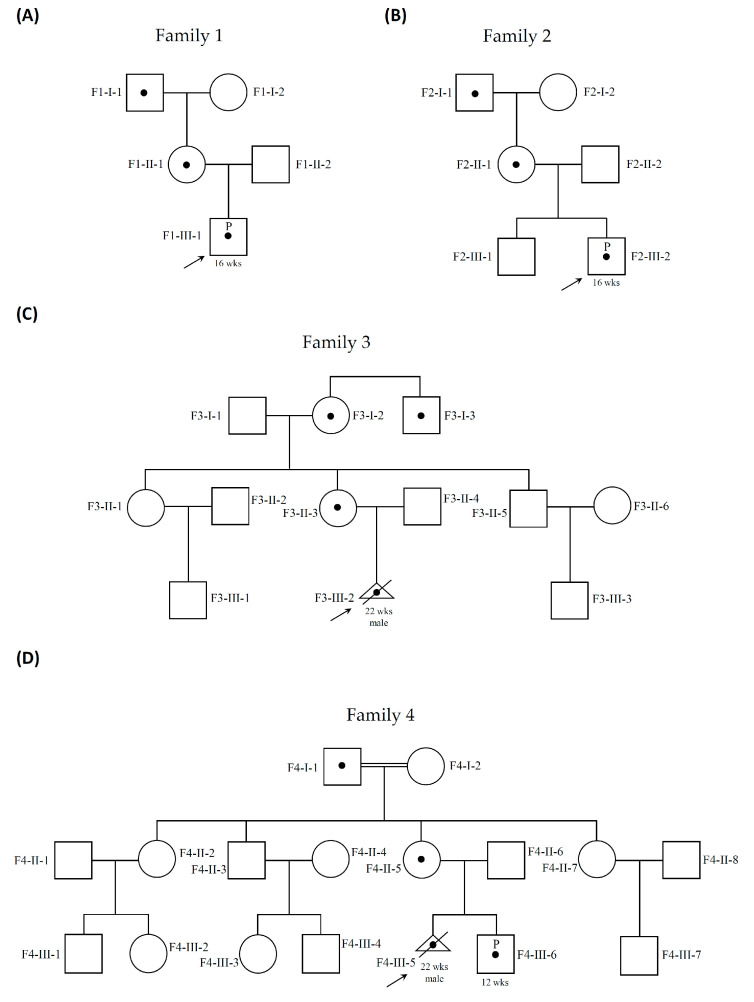
Pedigree information of four families involved chromosome number variations on X chromosome (X-CNVs) in male fetuses. (**A**) Family 1, (**B**) Family 2 and (**C**) Family 3 have X-CNVs on chromosome Xp22.31. (**D**) Family 4 has an X-CNV on chromosome Xq28. All the X-CNVs are traced back to at least one healthy male familial member in each family. Male indicated by square, female by circle, carrier by a dot in the middle of the symbol, pregnancy by a letter “P” in the middle of the symbol, termination of pregnancy by triangle with a slash, and the first case in a family seeking genetic testing by arrow. wks: weeks.

Four healthy women from four families (F1-II-1, F2-II-1, F3-II-3 and F4-II-5) and 14 of their familial members (F1-I-1, F1-I-2, F1-II-2, F2-I-1, F2-I-2, F2-II-2, F3-I-1, F3-I-2, F3-I-3, F3-II-4, F3-II-5, F4-I-1, F4-I-2 and F4-II-6) were enrolled in this study (Figure 1). These women visited our hospital for genetic counseling of their singleton male pregnancies during 2017 to 2019. The women underwent amniocentesis in the second trimester due to advanced maternal age (AMA) or a high risk of trisomy 21. CMA disclosed cryptic X-CNVs in fetuses of the four women (Table 1). Three fetuses had various sizes of microduplication at Xp22.31 (F1-III-1, F2-III-2 and F3-III-2), and the remaining one presented a microduplication about 431 kb in size at Xq28 (F4-III-6). Parental follow-up analyses revealed that all the X-CNVs were inherited from the healthy mothers. The X-CNVs were classified as VUS or associated with X-link intellectual disability (ID), according to the available databases and in-house database, and published literature. Actually, in the woman F4-II-5, the Xq28 microduplication were detected in her two continuing pregnancies (F4-III-5 and F4-III-6) (Figure 1). She had chosen late termination elsewhere in the first pregnancy (F4-III-5) due to the Xq28 microduplication before the visit of our hospital (Figure 1). Skewed XCI in the four heathy women carriers (F1-II-1, F2-II-1, F3-II-3 and F4-II-5) was excluded by analyzing the methylation status of the androgen receptor [5]. To assess the possible pathogenicity of the X-CNVs, segregation analysis by CMA was extended to other relatives in the maternal lineages, particularly the male relatives.

### 2.2. DNA Extraction

Peripheral blood samples were collected in EDTA anticoagulant tubes from the participants. The DNA was extracted using Puregene Extraction Kit (Qiagen, Hilden, Germany). The values and ratio of the absorbances at 260 nm and 280 nm were used to assess the DNA quality and purity using ND-1000 spectrophotometer (Labtech International, East Sussex, UK).

### 2.3. Chromosome Microarray Analysis (CMA)

The CMA was performed using oligonucleotide 8x60K CytoScan^®^ gene chip (Agilent customer design ID 040427, Changhua Christian Hospital, Changhua, Taiwan). DNA labeling and hybridization were carried out according to manufacturer’s recommendation. Scanned images were analyzed by Feature Extraction 9.5.3 software (Agilent Technologies, Santa Clara, CA, USA), and the extracted data were processed using the Agilent Genomic Workbench 7.0 program (Agilent Technologies, Santa Clara, CA, USA). In Families 1–4, the maternal grandparents of the fetuses were enrolled for CMA. In Family 3, the maternal uncle and the maternal granduncle were also included into analyses (Figure 1). The CMA findings were described based on the reference genome version of GRCh37. Online publicly available databases used for evaluation of the clinical significance of CNVs include DECIPHER (https://decipher.sanger.ac.uk/), NCBI dbVar’s nstd102 (Clinical Structural Variants) (http:// https://www.ncbi.nlm.nih.gov/dbvar/studies/nstd102/), European Cytogeneticists Association Register of Unbalanced Chromosome Aberrations (ECARUCA) (http://www.ecaruca.net), Online Mendelian Inheritance in Man (OMIM) (http://https://omim.org/), a genome database at the University of California, Santa Cruz (UCSC) (https://genome.ucsc.edu/) and Database of Genomic Variants (DGV) (http://dgv.tcag.ca/).

## 3. Results

The CMA demonstrated that at least one healthy male relative of the maternal lineage possessed the same X-CNVs of fetuses in each of the four families (Table 1). Three women under pregnant (F1-II-1, F2-II-1 and F4-II-5) opted to continue their current pregnancies. The remaining one woman who was seeking in vitro fertilization (IVF) with preimplantation genetic testing (PGT) for the familial X-CNV (F3-II-3) decided to choose natural conception. In follow-up with the three pregnant women at least one year later, all the born male babies with X-CNVs developed well without ID expression. The clinical histories of the women from Family 1–4 are detailed below.

### 3.1. Family 1

**Figure 2:**
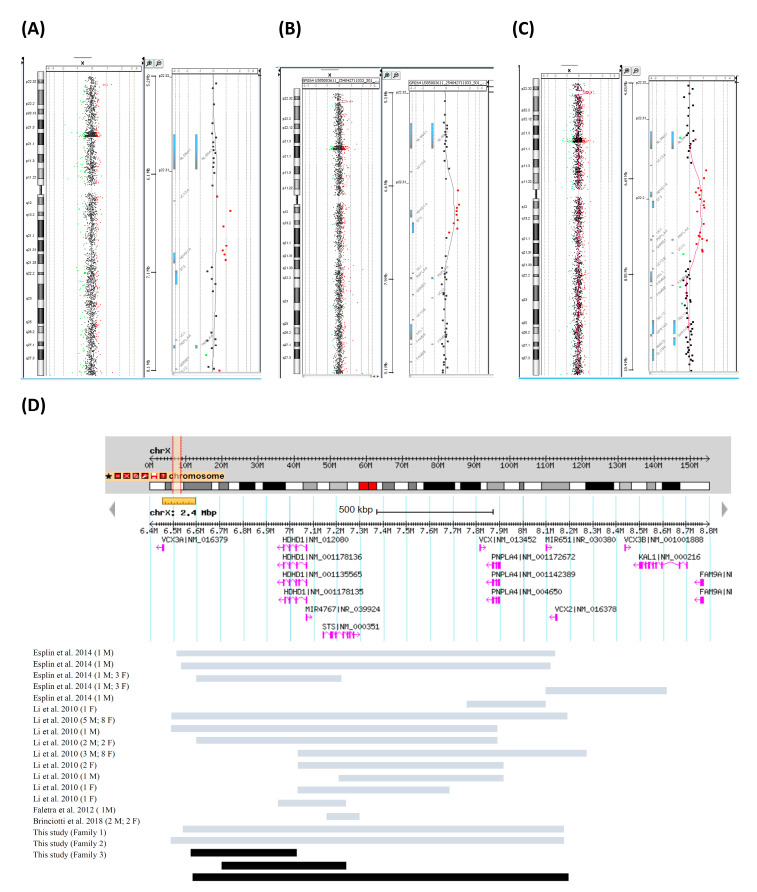
The Xp22.31 microduplications detected in the male fetuses of (**A**) Family 1 (F1-III-1: arr[GRCh37] Xp22.31(6552712_7033316)×2mat), (**B**) Family 2 (F2-III-2: arr[GRCh37] Xp22.31(6705268_7218859)×2mat), and (**C**) Family 3 (F3-III-2: arr[GRCh37] Xp22.31(6552712_8115153)×2mat). (**D**) Comparison of the breakpoints and genes included in Xp22.31 microduplications between our cases and the available published data. Previously reported cases indicated by gray bars and cases shown in this report indicated by black bars.

A 44-year-old woman (F1-II-1), G4P0A3, had a history of antiphospholipid syndrome. She underwent amniocentesis at 16 weeks (wks) of gestation age (GA) due to AMA. The cytogenetic results showed normal male karyotype 46,XY but CMA identified a microdeletion at 16p13.3 including *HBQ1*, *HBA1* and *HBA2* genes (arr[GRCh37] 16p13.3(215724_231196)×1) (15 kb) and a microduplication at Xp22.31 involving the *HDHD1*/*PUDP* gene (arr[GRCh37] Xp22.31(6552712_7033316)×2) (481 kb) (Table 1 and Figure 2A,D). One copy of 16p13.3 microdeletion including *HBA1* and *HBA2* was diagnosed to be a carrier of α thalassemia. The Xp22.31 microduplication was considered to be VUS. Parental follow-up analysis demonstrated the 16p13.3 microdeletion and Xp22.31 microduplication were of paternal and maternal origin, respectively. We advised the father of the pregnant woman (i.e., the maternal grandfather of the fetus) to share his genetic profile. CMA demonstrated the father harbored the Xp22.31 duplication (Table 1). By analyses of the three generations, it was concluded that the Xp22.31 duplication originated from the asymptomatic maternal grandfather and thus was considered to be “likely benign” in this family. Detailed ultrasound examinations revealed no adverse findings in the fetus. After a nondirective genetic counseling, the woman continued the pregnancy and finally gave birth to a 2675-g male via cesarean section at GA = 37 weeks and six days. At the time of submission, the baby had developed normally without ID feature for 23 months.

### 3.2. Family 2

A 28-year-old woman (F2-II-1), G2P1, underwent amniocentesis at GA = 16 wks due to increased nuchal fold in the first trimester. The fetal karyotyping was 46,XY but CMA revealed a microduplication at Xp22.31 encompassing *HDHD1*/*PUDP* and *STS* genes (arr[GRCh37] Xp22.31(6705268_7218859)×2) (514 kb) (Table 1, and Figure 2B,D). The Xp22.31 microduplications including *STS* gene were associated with X-linked ID and other abnormalities such as developmental delay, behavior problems, hypotonia and seizures although similar variations were also found in healthy human populations [8,9,10]. By tracing back to the parents, the Xp22.31 microduplication was demonstrated to inherit from the mother. We expanded the genetic analysis to the maternal grandparents and demonstrated that the Xp22.31 microduplication originated from the healthy grandfather. The woman chose to continue the pregnancy and gave birth to a 3425-g infant at GA = 38 wks and five days via vaginal delivery. The baby was 18 months old and had developed normally at the time of submission.

### 3.3. Family 3

A 35-year-old woman (F3-II-3), G1P0, visited our clinic seeking IVF with PGT. She had received amniocentesis elsewhere for karyotyping and CMA due to AMA. The cytogenetic result showed 46,XY but CMA revealed a likely pathogenic microduplication at Xp22.31 that covers five genes including *STS* (arr[GRCh37] Xp22.31(6552712_8115153)×2) (1562 kb) (Table 1 and Figure 2C,D) and is associated with X-linked ID and other abnormalities [8,9,10]. Parental analysis showed that the Xp22.31 microduplication is of maternal origin. Although features of incomplete penetrance were reported for the Xp22.31 microduplication, the pregnant woman finally chose termination of pregnancy (TOP) at GA = 22 wks. Before starting the assisted fertilization, we advised extended genetic analysis of the familial members of the woman. Her parents and younger brother (i.e., the maternal grandparents and uncle of the aborted fetus) were then enrolled for pedigree analysis. The Xp22.31 microduplication was detected in the mother of the woman. The woman subsequently asked an uncle on her mother’s side (i.e., a maternal granduncle of the aborted fetus) to accept CMA. The uncle was clinically normal and found to carry this Xp22.31 microduplication, which rendered that PGT was not necessary for this family. After a nondirective genetic counseling, the woman decided to choose natural conception instead of IVF with PGT.

### 3.4. Family 4

**Figure 3:**
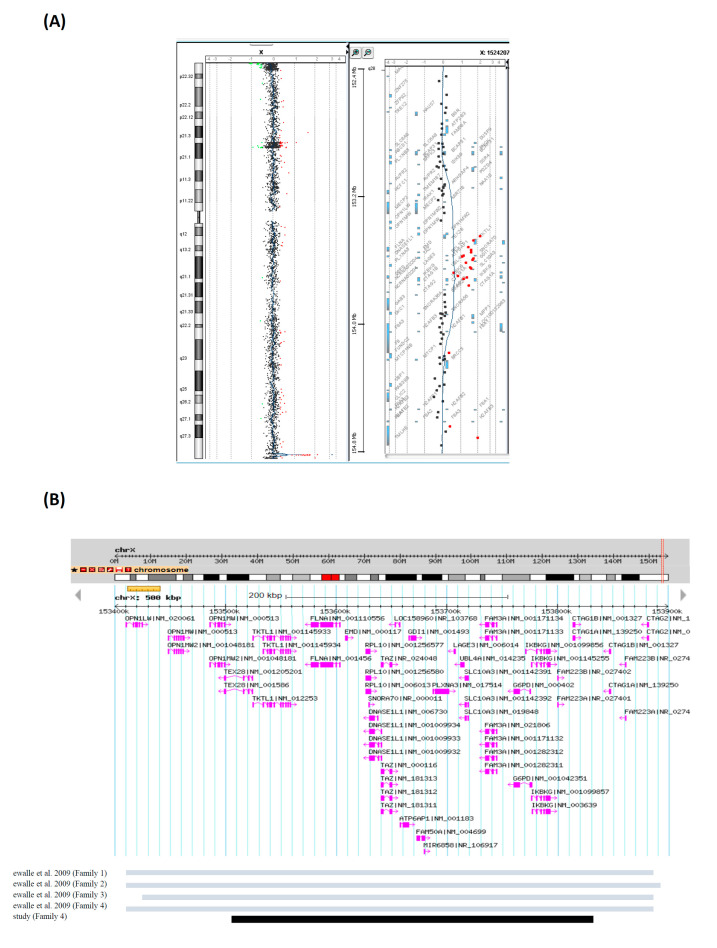
The Xq28 microduplication detected in the male fetus of (**A**) Family 4 (F4-III-6: arr[GRCh37] Xq28(153505485_153822717)×2mat). (**B**) Comparison of the breakpoints and genes included in Xq28 microduplications between our case and the available published data. Previously reported cases indicated by gray bars and the case shown in this report indicated by black bar.

A 33-year-old woman (F4-II-5), G2P0, visited our clinic seeking IVF with PGT since she had a pregnancy history of fetal X-CNV. During her first pregnancy, she received amniocentesis elsewhere due to a high risk of trisomy 21 by a maternal serum screening. The cytogenetic analysis showed a normal male karyotype 46,XY but CMA detected a Xq28 microduplication that included 22 genes (arr[GRCh37] Xq28(153505485_153822717)×2) (317 kb) (Table 1 and Figure 3). Parental follow-up analysis showed the Xq28 microduplication inherited from the healthy mother. The male fetus was considered to be at high risk of X-linked ID and recurrent infection because similar Xq28 microduplications had been associated with abnormal phenotypes (chromosome Xq28 microduplication syndrome, OMIM #300815) [11]. The woman opted for TOP at GA = 22 wks. During visits, a linkage analysis was designed for the woman to achieve PGT. After one cycle of failed IVF, the woman conceived spontaneously. Chorionic villus sampling was performed at GA = 12 wks. The fetus showed a normal male karyotype 46,XY but also carried the maternal Xq28 microduplication. We advised the father of the pregnant woman to undergo CMA. Unexpectedly, the X-CNV was found to have been transmitted from the maternal grandfather who is healthy and asymptomatic. After a nondirective genetic counseling, the woman chose to continue the pregnancy. The antenatal course was uneventful except for the finding of mega cisterna magna in the third trimester. She gave birth to a 2450-g male fetus at GA = 40 wks vaginally. The baby was 17 months old and had developed normally at the time of submission.

## 4. Discussion

The rapid accumulation of CMA data enables us to integrate large numbers of CNVs across a wide range of patients to access more orphan diseases, which were considered to be puzzles in the past. On the other hand, how to assess the clinical consequence of some CNVs remains a difficult task because a significant number of CNVs are found in normal populations [2,12,13]. Application of CMA to in utero fetuses instead of postnatal phenotypically abnormal individuals faces a much more difficult circumstance. First, among the diverse CNVs may be detected prenatally, not all CNVs are associated with adverse outcomes. Typically, the interpretation of possible clinical consequence of CNVs relies on cases with similar CNVs reported in the literature and latest public databases. Second, most of the reported genotype-phenotype associations of CNVs are based on postnatal studies, specifically the reference databases come from affected subjects, providing a bias inference. Moreover, lots of abnormal phenotypes such as hypotonia, developmental delay, and ID cannot be noted until infancy. These discrepancies render the prenatal interpretation of CNVs challenging.

A five-tier system has been proposed by the American College of Medical Genetics (ACGM) for variant classification. According to this system, CNVs can be classified into pathogenic, likely pathogenic, uncertain significance, likely benign, and benign [14]. The classification of variants as pathogenic, likely pathogenic, likely benign, and benign undergo extensive review. However, it should be noted that at present a significant number of CNVs do not have enough evidence to support being placed in any tier except for the uncertain significance. This is because of the heterogeneous nature of most diseases. Under certain circumstances, the clinical sequences of some CNVs may depend on a set of other factors such as ethnical background, gene-gene interactions based upon different genomic backgrounds or even environmental influence [3].

Assessment of the phenotypic relevance of X-CNVs is relatively complex because it involves X-inactivation in women and lack of homologous genes as a backup on the chromosome X in men, rendering male are more susceptible to X-linked illness, such as ID. Actually, the causality between X-CNVs and male ID remains elusive. It is possible that the X-inactivation can vary in different females and even in different tissues of a female. However, it is difficult to devise a standard genetic testing to discern if CNVs are located on the silenced chromosome X in females. Otherwise, it is also possible that the database of co-segregation tests for X-CNVs in different genders is relatively too small since it is not feasible to include as many family members as possible in the pedigree analyses. In a postnatal setting, Willemsen et al. [7] examined a large cohort of 4077 male and female patients who were identified to carry X-CNVs. They set a model of interpretation of the clinical attributes of each CNV by well-defined criteria and previous literature. The model included inheritance pattern (de novo, maternal or paternal origin), X-inactivation, occurrence in patients and controls, CNV size, and gene content, whether there was an association with cognitive disorder or congenital anomaly, and presence of brain expression and function, as well as the phenotypes in mouse models. They categorized the CNVs into three groups (likely pathogenic, unknown relevance and likely non-pathogenic), and the prevalence rate of X-CNVs was about 1.3%, of which 0.3% was interpreted as (likely) pathogenic [7]. Furthermore, another cohort survey of 2222 sporadic male patients with ID disorder aimed to determinate the frequency and nature of X-CNVs. They sorted the CNVs by criteria similar to Willemsen et al. [7] and only 19 males with ID (0.85%) were considered to carry likely pathogenic X-CNVs [6].

In our study, Families 1, 2, and 3 have had various sizes of microduplication at Xp22.31. The Xp22.31 region is prone to genomic instability due to non-allelic homozygous recombination and the clinical significance of Xp22.31 microduplication remains controversial. In Family 1, the duplicated segment only encompassed the *HDHD1/PUDP* gene which encodes pseudouridine-5′-phosphatase and is often deleted in X-linked ichthyosis [15]. In Family 2, the duplicated segment included the *STS* and *HDHD1/PUDP* genes. Chromosome Xp22.31 duplication including *STS* has been recognized as a pathogenic CNV by multiple authors [8,16]. In Family 3, the size of the duplicated region was larger and harbored more genes; it consisted of *HDHD1/PUDP*, *STS*, *VCX*, *PNPLA4*, and *MIR651*. Li et al. [10] analyzed 29 individuals with various sizes of Xp22.31 duplication that included the *STS* gene as part of 7793 CMA samples from five countries: Australia, France, United Kingdom, United States, and Germany. The estimated frequency of Xp22.31 duplication in the healthy control population is 0.15%, compared to 0.37% in a cohort of individuals with an abnormal phenotype [10]. Since the segment detected in Family 3 overlapped what was discussed in the study, the woman (F3-II-3) chose TOP in her first pregnancy. However, further published literature noted that similar Xp22.31 duplications recurred in some families in not only phenotypically abnormal but also normal individuals [8,9,17]. As a result, the Xp22.31 duplication may be simply a benign CNV or a predisposing factor (i.e., a high-risk locus). However, genetic mechanisms such as incomplete penetrance and variable expressivity that can case variable phenotype cannot be excluded. Further evidence for clarification of the clinical significance of the Xp22.31 duplication is therefore needed.

In Family 4, an Xq28 microduplication was identified. The Xq28 region is also prone to genomic instability since low copy repeats result in micro-rearrangement. Diseases caused by Xq28 duplications can be classified into three categories according to the genes involved: *MECP2* (OMIM 300005) [18], *GDI1* (OMIM 300815) [10], and *RAB39B* (int22h1/int22h2-mediated Xq28 duplication syndrome) [19]. Vandewalle et al. [10] reviewed four families with X-linked ID and identified a 0.3 Mb copy number gain distal to *MECP2*, which included 18 annotated genes. The *GDI1*, a gene expressed in the brain, and *IKBKG*, a gene encoded a protein essentially in the NF-kB signaling that controls several cellular and developmental processes, were considered to be the causative genes in the region [11,20]. The fetus in Family 4 (F4-III-6) almost overlapped the segment and involved both of *GDI1* and *IKBKG*. Therefore, we supposed that the Xq28 duplication would lead to abnormal phenotype during the first session of genetic counseling. The pregnant woman (F3-II-5) finally chose TOP. Since the woman (F3-II-5) sustained pregnancy with recurrent Xq28 microduplication, we extended the analyses to her cognitively normal father and demonstrated that he was a carrier but asymptomatic. This result leads to suggest that the previous genome-wide CMA study did not correlate the genetic dosage change to the abnormal phenotype or alternatively other coexistent factors that alter the genetic burden in the family.

**Figure 4:**
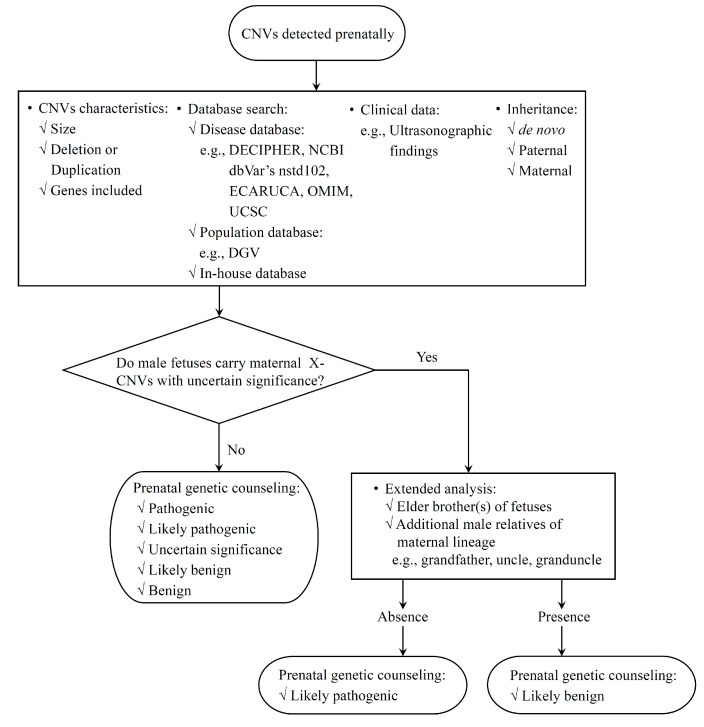
The flowchart of prenatal interpretation of clinical consequence of CNVs. An extended analysis including other male relatives from the maternal lineage is suggested for the prenatal setting in which male fetuses carry X-CNVs from unaffected mothers. Maternally inherited X-CNVs identified in male fetuses that are also found in other male relatives of the maternal lineage are tentatively considered to be likely benign.

For clinical reference, we provide a flowchart for the interpretation of X-CNVs (Figure 4); in it the gender of the fetus must be taken into consideration when it comes to X-CNVs. Moreover, expanding segregation analysis by including more male relatives in the maternal lineage is essential in situations where male fetuses inherited X-CNVs from their healthy mothers and genetic counseling is offered. However, a nondirective counseling remains the golden standard since the causality may be verified with the application of new methodologies or databases being amended with increasing experience. For example, Qiao et al. [21] carried out exome sequencing to identify a de novo *PURA* mutation on a male proband of familial Xq22.31 microduplication including *VCX* and *PNPLA4*, which was transmitted from his cognitively normal maternal grandfather. The presence of healthy male relatives could reassure the pregnant women with X-CNVs before they opt for TOP, such as Families 3 and 4 in this report. However, risks such as incomplete penetrance and variable expressivity should be emphasized in prenatal counseling.

## 5. Conclusions

In the past few years, there has been a significant increase in the diagnostic yield of rare diseases by CMA, but prenatal interpretation of CNVs, particularly X-CNVs, remains challenging. As was showed in this study, some reported X-CNVs previously recognized as VUS or even pathogenic can be found in healthy male individuals, indicating a phenotypic heterogeneity of CMA variants. Therefore, for male fetuses carried X-CNVs from their healthy mothers, we highlight that an extended study, in addition to the trio analysis, including male relatives of the maternal lineage provides additional information for prenatal counseling. However, we admit that the sample size of this study is limited and phenomena such as incomplete penetrance and variable expressivity of X-CNVs cannot be excluded. Large case studies include more rigorous clinical approaches, in addition to experimental and statistical databases, to understand the molecular mechanisms related to X-CNVs (e.g., genotype-phenotype correlation) are still necessary.

## Figures and Tables

**Table 1:** Summary of the copy number variations at the chromosome X (X-CNVs) prenatally detected in male fetuses of four families (Family 1–4).

Family	Fetus ^1^	X-CNV	Size (kb)	Genes Included	Inheritance	Healthy Male Relatives (Maternal Lineage) with the X-CNV ^1^
1	F1-III-1	1. arr[GRCh37] 16p13.3(215724_231196)×1 2. arr[GRCh37] Xp22.31(6552712_7033316)×2	1. 15 2. 481	1. *HBQ1, HBA1, HBA2* 2. *HDHD1*/*PUDP*	1. Paternal 2. Maternal	F1-I-1
2	F2-III-2	arr[GRCh37] Xp22.31(6705268_7218859)×2	514	*HDHD1*/*PUDP*, *STS*	Maternal	F2-I-1
3	F3-III-2	arr[GRCh37] Xp22.31(6552712_8115153)×2	1562	*HDHD1/PUDP, STS, VCX, PNPLA4, MIR651*	Maternal	F3-I-3
4	F4-III-6	arr[GRCh37] Xq28(153505485_153822717)×2	317	*TEX28, TKTL1, FLNA, EMD, RPL10, SNORA70, DNASE1L1, TAZ, ATP6AP1, GDI1, FAM50A, PLXNA3, LAGE3, UBL4A, SLC10A3, FAM3A, G6PD, IKBKG, NCRNA00204B, NCRNA00204, CTAG1B, CTAG1A*	Maternal	F4-I-1

^1^ The designations accord with Figure 1.
